# Psoas Abscess—Treatment of, with Sulphurous Acid

**Published:** 1873-02

**Authors:** Henry Manfred


### PSOAS ABSCESS—TREATMENT OF, WITH SULPHUROUS ACID.

BY HENRY MANFRED, M.D.

The medical world in every age brings to light new discoveries and increased appliances for the use and healing of mankind; these discoveries are often forced upon physicians by the painful emergencies of the hour, and drive them from the beaten track into new and untried paths, which oftentimes, perhaps, end in disappointment, but sometimes result in new and unexpected success. By such means, men often unconsciously become discoverers: Our generation has witnessed the introduction of chloroform and sulphuric ether as anæsthetics, which have robbed surgery of many of its horrors, and still later of carbolic acid and its allies (sulphocarbolates), which have enabled the practitioner to treat with success and satisfaction many injuries and diseases that had heretofore too often bid defiance to all known remedies, even when administered with well-directed judgment and skill.

*Sulphurous acid* has long been used by wine manufacturers to sweeten old and fusty wine barrels, which are thereby rendered instantaneously as sweet and wholesome as new ones; and the method of burning sulphur, thereby forming sulphurous acid, is known to many medical men as the best means of disinfecting bedclothes, rooms and wearing apparel in cases of malignant variola; this is a method which I have always found so effective, that I rarely use any other disinfectant. But the credit of first introducing sulphurous acid as a remedial agent in *incised wounds* and *ill-conditioned ulcers* is rightfully due to Dr. James Dewar, Kirkcaldy, Scotland, who is the author of two excellent suggestive papers upon "Sulphurous Acid Fumigation," *Braithwaite's Retrospect*, 1868, page 33, vol. 56; and again upon "Sulphurous Acid as applied to

Wounds and Sores," page 127, vol. 56, of the same year. The published reports of his cases, in 1868, induced me to make similar experiments, and with the most satisfactory and oftentimes astonishing results, in incised, contused and lacerated wounds; in follicular laryngitis (clergyman's sore throat). Professor Syme also gives a case in which he used the acid as a primary dressing to a severe wound, and was greatly surprised, at his second visit, to find it in such a remarkable state of forwardness. I can fully corroborate all that Dr. Dewar has said about it, and more too. It has, undoubtedly, *unrivaled disinfecting power, far superior to carbolic acid.* It is not like the former, a powerful irritant; besides, being altogether free from its disgusting and penetrating smell. But the most remarkable characteristic of this acid is its *antagonism* to *pus,* the great bugbear of surgeons, and its use enables them to bid defiance to its destructive ravages, which have heretofore slain so many millions, both in military and civil life. My success in these cases induced me to extend its use to others not before thought of. In 1868, soon after reading the papers referred to, I was called to see a little girl, four years old, who had been under the care of another doctor, and was suffering from symptoms resembling *coxalgia.* She had been affected with a scrofulous discharge from the ear, for two years, which had suddenly disappeared, and was just recovering from an attack of pneumonia, when these phenomena presented themselves. The little patient, who was a most engaging, interesting child, complained constantly of severe pain in the left hip and leg, which she kept constantly flexed, and would scream out at the slightest attempt being made to straighten the limb. The child had lost both health and strength, and was suffering from irritative fever, accompanied with great restlessness. She was placed upon iron tonics, with a liberal diet, to improve her circulation, and recruit her waning powers. After an interval of ten days, a lumbar abscess on a line with the last lumbar vertebræ of the left side, declared itself. I opened the abscess early, according to the Liston method, using carbolic acid freely, when nearly half a pint of ill-conditioned pus flowed out, rendering the room very offensive. The abscess was carefully closed, and a small tent inserted to obviate the second use of the knife. The discharge of pus continued the following, next, and succeeding days as bad as ever. I used ferri mur. dil. carbolic lotion, Condy's fluid, chloride zinc, sulphate of zinc. The cavity of the abscess was injected with these astringents, successively used, each and all in their turn, to be exchanged for others when found inefficient. Sometimes I thought that I had at last got the right remedy, which would control the pus-secreting surface, but at my next visit the pus flowed more copiously than ever; the vital powers were ebbing fast from this continued drain, which had lasted some weeks, and, if not stopped, the patient must soon sink beyond recall. Whenever I had closed the wound with plaster and bandages, in order to procure adhesion of its walls, irritative fever, loss of appetite, and restless nights from absorption of the poisonous secretions; upon opening it again, these ceased, but the large daily drain that followed would certainly and speedily kill my little patient. I found myself placed between Scylla and Charybdis. Death appeared near and certain; threatened on the one side from the absorption of the poisonous *materies morbi*, and upon the other from the asthenia and exhaustion, which would certainly result if it was allowed to drain away. The second evil was perhaps the least, but both were bad enough. Sulphurous acid had often before stood my firm friend when in need, and I determined to try it now. I was not, at that time, acquainted with its *great sanitive* power, as I am now, and resorted to its use not without some misgiving as to the final issue. But something had to be done, and that speedily. I injected the cavity thoroughly with the acid, and was gratified to find that the discharge had materially lessened; by the following day, and after three days, had almost entirely ceased; the abscess gradually closed by granulation, the patient rallied, and in less than a month had quite recovered, and is now in perfect health. The result exceeded my most sanguine expectations, and quite astonished the family and neighborhood, who had regarded the case as irretrievably hopeless. The case had lasted five months. Four years have now elapsed, and the child's health continues excellent. Nor is there now the slightest deformity or shortening of the limb to be seen. I have since used sulphurous acid in *confluent* and *simple variola*, in *scarlatina*, in *typhoid fever*, in *multiple abscess*, in *adynamic fevers*, and in those cases of blood poisoning where the secretions are offensive, the vital powers declining, accompanied with a general tendency to putrescency and decay. In variola, I generally prescribe half a drachm of the acid diluted with water, three or four times a day, and apply it also locally to the pharynx, by means of a gargle or atomizer. Under its use the offensive odor, so peculiar to the disease, is greatly lessened or disappears altogether, and the irritability and heat of the suppurating surface, greatly diminished, affording much relief to the patient. In the bowel lesion of typhoid fever, the writer has found its internal use highly beneficial; it lessens the acridity of the discharge, which it disinfects, thereby preventing its poisonous absorption, while it promotes the healing of the ulcers themselves, very much in the same manner as when applied externally to wounds; in short, its unrivaled sanative power in such cases as have been mentioned may be summed up in three words—*tuto cito et jucunde.*—*Cincinnati Lancet and Observer.*

